# Identification of pathogenic genes in *Campylobacter jejuni* isolated from broiler carcasses and broiler slaughterhouses

**DOI:** 10.1038/s41598-021-84149-1

**Published:** 2021-02-25

**Authors:** Yuli Melisa Sierra-Arguello, Gustavo Perdoncini, Laura Beatriz Rodrigues, Luciana Ruschel dos Santos, Karen Apellanis Borges, Thales Quedi Furian, Carlos Tadeu Pippi Salle, Hamilton Luiz de Souza Moraes, Marcos José Pereira Gomes, Vladimir Pinheiro do Nascimento

**Affiliations:** 1 Centro de Diagnóstico e Pesquisa em Patologia Aviária, Faculdade de Veterinária, Universidade Federal do Rio Grande do Sul, Av. Bento Goncalves 8824, Porto Alegre, Rio Grande do Sul CEP 91540-000 Brazil; 2 Faculdade de Veterinária, Universidade de Passo Fundo, Passo Fundo, Brazil; 3 Laboratório de Bacteriologia Veterinária, Faculdade de Veterinária, Universidade Federal do Rio Grande do Sul, Porto Alegre, Brazil

**Keywords:** Bacterial pathogenesis, Infectious-disease epidemiology

## Abstract

*Campylobacter jejuni* is one of the most common causes of foodborne diseases worldwide. There are few reports on *Campylobacter* strains isolated from Latin-American countries. Here, 140 *C. jejuni* strains isolated from cloacal and transport boxes swabs, water from chiller tanks, and broiler carcasses of five poultry companies in Southern Brazil were identified using phenotypic and genotypic methods. Polymerase chain reaction (PCR) was used to analyze eight *C. jejuni* virulence markers: *fla*A, *cad*F, and invasion-associated (*iam*) genes, *cdt*ABC operon (associated with the cytolethal distending toxin), and plasmidial *vir*B11 and *wla*N genes were present in 78.5%, 77.8%, 0%, 74.2%, 22.1%, and 10.7% of samples, respectively. There were 25 different virulence profiles: 1 (*cdt*A*, cdt*B*, cdt*C*, fla*A*,* and *cad*F), 2 (*cdt*A*, cdt*B*, cdt*C*, fla*A*, cad*F*,* and *vir*B11), and 3 (*cdt*A, *cdt*B*, cdt*C*, fla*A, *cad*F*,* and *wla*N) were the most common (> 60% of strains). We provide insight into factors related to the occurrence of this pathogen and their epidemiology.

## Introduction

*Campylobacter* spp. are widespread in nature and are commensal organisms, present in the gut of many birds and domestic animals. However, campylobacteriosis is a globally distributed disease that has a significant impact on public health^1–3^. Currently, there are 17 species and six subspecies assigned to the genus *Campylobacter*, of which the most frequently reported to cause human diseases are *C. jejuni* (subspecies *jejuni*) and *C. coli*^3^. Infections by *C. jejuni* are one of the most common causes of foodborne diarrheal illness in humans^1–3^. Other clinical presentations of *Campylobacter* infection include meningitis, septicemia, localized extraintestinal infections, reactive arthritis or Reiter’s syndrome^4,5^, and immune-reactive complications, such as Guillain–Barré Syndrome (GBS), and its variant, Miller Fisher Syndrome (MFS)^6,7^.

*Campylobacter* can enter the food chain of humans in different ways, and poultry products are considered the most important source of *C. jejuni* infection in humans^2,8,9^. The development of the poultry industry, due to the improvements obtained in the genetics of birds, and also in the nutrition and management systems, enables a large-scale poultry production. However, high density and confinement systems favor the introduction and spread of pathogens. Human infection may occur by consumption of raw or undercooked poultry, or by cross-contamination with *C. jejuni* from these products^2,10–12^. The slaughter stages that are considered critical contamination points are scalding, plucking, evisceration, washing, and chilling^12^.

Despite its public health importance, virulence factors and mechanisms of *C. jejuni* pathogenesis remain poorly studied in Latin-American countries^13^, especially regarding their occurrence among poultry companies and the different sources of isolation throughout the slaughtering process. The majority of virulence factors are associated with motility, chemotaxis, adhesion and colonization of intestinal epithelial cells, invasion and translocation capabilities, production of toxins and secreted proteins, and other mechanisms essential for bacterial survival^14,15^. Other pathogenic mechanisms, such as the production of enterotoxins and cytotoxins, and the ability to adhere and invade epithelial cells, have been proposed to play a role in enteritis^16,17^. The cytolethal distending toxin (CDT) holotoxin consists of three subunits encoded by the genes *cdt*A, *cdt*B, and *cdt*C, which are genetically arranged as an operon^18,19^. The expression of all three *cdt* genes is required for maximum toxin activity^20^. The *cdtA* and *cdtC* gene products are two subunits responsible for toxin binding to the cell membrane and for the delivery of the *cdtB* gene product (CdtB), which is the enzymatically active subunit^14^.

*Campylobacter* exhibits bipolar flagella composed of a hook–basal body complex, which is composed of several different proteins, and the extracellular filament structural components, which are composed of a major flagellin protein, FlaA (coded by *flaA*), and a minor flagellin protein, FlaB (coded by *flaB*)^14^. The *fla*A gene seems to be highly conserved among *Campylobacter* isolates, and its transcription level is usually higher than that of *fla*B^11,21^. Thus, flagella are crucial for the attachment to intestinal epithelial cells and are involved not only in motility and chemotaxis, but also in the secretion of virulence proteins, autoagglutination, microcolony formation, and avoidance of the innate immune response^22^. The *cad*F (*Campylobacter adhesion to fibronectin F*) gene encodes an adhesion and fibronectin-binding protein involved in the process of invasion, influencing the microfilament organization in host cells^14,23^. Another virulence gene linked to *Campylobacter* invasiveness is the *invasion-associated marker* (*iam*) gene^24^. It has been suggested that the pVir plasmid, which encodes for proteins of a type IV secretion system, and the mutation of the plasmid *vir*B11 gene results in a reduced adherence and invasion potential in vitro, as well as in less severe symptoms in vivo^25^. The *wlaN* gene is presumably involved in the expression of ganglioside mimics in Guillain–Barré Syndrome and may encode β-1,3-galactosyltransferases with identical enzymatic activities^26,27^.

Considering that *Campylobacter* may be transferred from animals to humans, and that poultry is its main reservoir^2,8^, it is important to know the genetic profile of circulating strains in broiler populations and whether all *Campylobacter* isolates obtained from different companies and sources of isolation have the same virulence potential. In addition, many aspects concerning epidemiology, public health significance, and risk assessment studies are recent and still poorly known in Brazil, and there are no Brazilian legal standards established regarding *Campylobacter* presence or counts in food products. The purpose of this study was to determine whether eight genes previously identified as coding pathogenic markers in *Campylobacter* were present in the 140 samples obtained from several broiler sources and collected from five poultry companies using polymerase chain reaction (PCR).

## Results

Out of a total of 140 screened samples, all of them were confirmed as having *Campylobacter jejuni* species*.* This method is based on the detection of 16S rRNA and *map*A genes. The occurrence of the virulence factor genes *fla*A, *cad*F, *iam*, *vir*B11, *wla*N, and the gene cluster *cdt*ABC was analyzed, and the virulence profiles that were observed are described in Table 1. Distribution of *C. jejuni* strains has shown 25 different virulence profiles. Profiles 1 (*cdt*A*, cdt*B*, cdt*C*, fla*A*,* and *cad*F), 2 (*cdt*A*, cdt*B*, cdt*C*, fla*A*, cad*F*,* and *vir*B11), and 3 (*cdt*A, *cdt*B*, cdt*C*, fla*A, *cad*F*,* and *wla*N) were the most common, representing 30% (42/140), 20% (28/140), and 10.7% (15/140) of the strains, respectively. Together, these three profiles represented more than 60% of the strains. The distribution of profiles according to the poultry company is described in Table 1. For companies A, D, and E, the most common profile was 1, corresponding to 36%, 37.5%, and 80% of the strains, respectively. For company B, profile 2 was the most common (33%), and for company C, profiles 3 (26.4%) and 1 (24.5%) were the most common.

**Table 1:** Distribution of virulence profile among *Campylobacter jejuni* strains.

Profile number	Virulence profile	Distribution		Distribution according to the poultry company % (n)				
		n = 140	%	A (n = 25)	B (n = 15)	C (n = 53)	D (n = 42)	E (n = 5)
1	*cdt*A*, cdt*B*, cdt*C*, fla*A*, cad*F	42	30,0	36 (9)	6.7 (1)	24.5 (13)	35.7 (15)	80 (4)
2	*cdt*A*, cdt*B*, cdt*C*, fla*A*, cad*F*, vir*B11	28	20,0	16 (4)	33 (5)	11.3 (6)	30.9 (13)	0
3	*cdt*A*, cdt*B*, cdt*C*, fla*A*, cad*F*, wlaN*	15	10,7	0	0	26.4 (14)	0	20 (1)
4	*cdt*A*, cdt*B*, cdt*C*, cad*F	9	6,4	4 (1)	0	1.9 (1)	9.5 (4)	0
5	*cdt*A*, cdt*B*, cdt*C*, fla*A	6	4,3	4 (1)	0	5.7 (3)	9.5 (4)	0
6	*cdt*A*, cdt*B*, cdt*C	5	3,6	0	6.7 (1)	7.5 (4)	2.4 (1)	0
7	*cdt*A*, cdt*C*, fla*A*, cad*F	4	2,9	16 (4)	0	7.5 (4)	0	0
8	*cdt*B*, cdt*C	4	2,9	0	0	3.8 (2)	0	0
9	*cdt*A*, cdt*C	3	2,1	0	6.7 (1)	0	0	0
10	*cdt*A*, cdt*C*, cad*F	3	2,1	0	6.7 (1)	0	4.8 (2)	0
11	*fla*A	3	2,1	4 (1)	6.7 (1)	1.9 (1)	0	0
12	*cdt*A*, fla*A*, cad*F	2	1,4	4 (1)	6.7 (1)	0	0	0
13	*cdt*B*, cdt*C*, fla*A	2	1,4	4 (1)	0	1.9 (1)	0	0
14	*cdt*C*, fla*A	2	1,4	0	0	1.9 (1)	2.4 (1)	0
15	*cdt*C	2	1,4	0	6.7 (1)	0	2.4 (1)	0
16	*fla*A*, cad*F	1	0,7	0	0	0	2.4 (1)	0
17	*cdt*C*, fla*A*, cad*F	1	0,7	4 (1)	0	0	0	0
18	*cdt*A*, cdt*C*, fla*A*, cad*F*, vir*B11	1	0,7	0	6.7 (1)	0	0	0
19	*cad*F*, vir*B11	1	0,7	0	6.7 (1)	0	0	0
20	*fla*A*, cad*F*, vir*B11	1	0,7	0	6.7 (1)	0	0	0
21	*cdt*B*, cdt*C*, cad*F	1	0,7	0	0	1.9 (1)	0	0
22	*cdt*A*, fla*A	1	0,7	0	0	1.9 (1)	0	0
23	*cdt*A	1	0,7	4 (1)	0	0	0	0
24	*cdt*B	1	0,7	0	0	1.9 (1)	0	0
25	No virulence genes detected	1	0,7	4 (1)	0	0	0	0

The results showed that only one strain did not present any virulence markers. A total of 61.4% (86/140) of *C. jejuni* strains possessed five or six virulence determinants, 24.3% (34/140) presented three or four virulence*-*associated genes and the presence of one or two genes was detected in 13.6% (19/140) of isolates. No strain had more than six virulence markers.

The cytotoxin-encoding cluster *cdt*ABC was detected in 74.3% (104/140) of the isolates. The frequency rates found for *cdt*A, *cdt*B, and *cdt*C were 85% (119/140), 80% (112/140), and 92.1% (129/140), respectively. The *flaA* and *cadF* genes were found in 78.6% (110/140) and 77.9% (109/140) of the strains, respectively. The *virB11* and *wlaN* genes were significantly (*p* = 0.0001) less frequent, and were detected in 22.1% (31/140) and 10.7% (15/140) of strains, respectively. Moreover, *iam* was not detected in any of the investigated strains.

The PCR detection results of eight virulence-associated genes are described in Tables 2 and 3, according to the source and company from which they were isolated. The comparison among isolation sources has shown no significant differences, regardless the virulence gene (Table 2). Significant differences in gene frequency among the isolation companies were observed for *vir*B11 between companies B and C (*p* = 0.0013); in addition, *cdt*B were significantly less common in company B (*p* = 0.002) (Table 3).

**Table 2:** Distribution of virulence markers among *Campylobacter jejuni* strains, according to the source of isolation.

Source of isolation		Frequency (%) of positive of strains (n/N)								
		*fla*A	*cad*F	*cdt*A	*cdt*B	*cdt*C	*cdt*ABC	*iam*	*vir*B11	*wla*N
Cloacal swab (n = 5)		80 (4/5)^a^	80 (4/5)^a^	80 (4/5)^a^	100 (5/5)^a^	100 (5/5)^a^	60 (3/5)^a^	0	40 (2/5)^a^	0
Swab of broiler transportation cage (n = 3)		66.7 (2/3)^a^	100 (3/3)^a^	100 (3/3)^a^	66.7 (2/3)^a^	100 (3/3)^a^	66.7 (2/3)^a^	0	0	33.3 (1/3)^a^
Broiler carcasses through slaughter process (n = 115)	Scalding (n = 3)	66.7 (2/3)^a^	33.3 (1/3)^a^	66.7 (2/3)^a^	33.3 (1/3)^a^	33.3 (1/3)^a^	33.3 (1/3)^a^	0	0	0
	Plucking (n = 9)	100 (9/9)^a^	100 (9/9)^a^	100 (9/9)^a^	100 (9/9)^a^	100 (9/9)^a^	100 (9/9)^a^	0	33.3 (3/9)^a^	33.3 (3/9)^a^
	Evisceration (n = 7)	85.7 (6/7)^a^	71.4 (5/7)^a^	85.7 (6/7)^a^	85.7 (6/7)^a^	85.7 (6/7)^a^	85.7 (6/7)^a^	0	28.6 (2/7)^a^	0
	Spray-washing (n = 7)	71.4 (5/7)^a^	57.1 (4/7)^a^	71.4 (5/7)^a^	85.7 (6/7)^a^	100 (7/7)^a^	71.4 (5/7)^a^	0	0	28.6 (2/7)^a^
	Cooled carcasses (n = 49)	77.6 (38/49)^a^	79.6 (39/49)^a^	83.7 (41/49)^a^	75.5 (37/49)^a^	89.8 (44/49)^a^	69.4 (34/49)^a^	0	16.3 (8/49)^a^	12.2 (6/49)^a^
	Cooled cuts (n = 40)	80 (32/40)^a^	80 (32/40)^a^	92.5 (37/40)^a^	87.5 (35/40)^a^	97.5 (39/40)^a^	87.5 (35/40)^a^	0	32.5 (13/40)^a^	0
Chiller tank processing water (n = 17)		70.6 (12/17)^a^	70.6 (12/17)^a^	70.5 (12/17)^a^	70.6 (12/17)^a^	88.2 (15/17)^a^	52.9 (9/17)^a^	0	17.6 (3/17)^a^	17.6 (3/17)^a^

Different letters in the same column indicate significant differences (*p* < 0.005) among source of isolation (Fisher’s exact test; adjusted *p*-value).

**Table 3:** Distribution of virulence markers among *Campylobacter jejuni* strains, according to the poultry company.

Poultry company	Frequency (%) of positive of strains (n/N)								
	*fla*A	*cad*F	*cdt*A	*cdt*B	*cdt*C	*cdt*ABC	*iam*	*vir*B11	*wla*N
A (n = 25)	92 (23/25)^a^	84 (21/25)^a^	80 (20/25)^a^	60 (15/25)^a^	88 (22/25)^a^	56 (14/25)^a^	0	16 (4/25)^a,b^	0
B (n = 15)	66.7 (10/15)^a^	73.3 (11/15)^a^	73.3 (11/15)^a^	46.7 (7/15)^b^	73.3 (11/15)^a^	46.7 (7/15)^a^	0	53.3 (8/15)^b^	0
C (n = 53)	71.7 (38/53)^a^	71.7 (38/53)^a^	83 (44/53)^a^	90.6 (48/53)^a^	94.3 (50/53)^a^	77.4 (41/53)^a^	0	11.3 (6/53)^a^	26.4 (14/53)^a^
D (n = 42)	81 (34/42)^a^	81 (34/42)^a^	92.9 (39/42)^a^	88.1 (37/42)^a^	97.6 (41/42)^a^	88.1 (37/42)^a^	0	31 (13/42)^a,b^	0
E (n = 5)	100 (5/5)^a^	100 (5/5)^a^	100 (5/5)^a^	100 (5/5)^a^	100 (5/5)^a^	100 (5/5)^a^	0	0	20 (1/5)^a^

Different letters in the same column indicate significant differences (*p* < 0.010) among poultry companies (Fisher’s exact test; adjusted *p*-value).

## Discussion

The purpose of this study was to determine the presence of *C. jejuni* virulence markers as indicators of the potential role of these strains in the pathogenesis of human diseases. An understanding of the epidemiology of all foodborne zoonotic agents is essential for the implementation of control strategies and interventions^28^. Phylogenetic studies have demonstrated a large heterogeneity among isolates of the same species, with the absence of clones, indicating a high level of diversity of circulating genotypes^29^. Previous studies have demonstrated the increased pathogenic potential of *Campylobacter jejuni* isolates from Brazilian poultry during the last decade^30^. In the present study, the identification and frequency of this pathogen throughout the slaughter line revealed potential virulence-associated markers, emphasizing the zoonotic risk of avian-derived *Campylobacter* strains.

Similar to previous reports^31^, the *fla*A gene presented a high frequency (78.6%) in this study*.* The bacterial flagellum and the virulence-associated injectisome are complex and structurally related to nanomachines that bacteria use for locomotion or the translocation of virulence factors into eukaryotic host cells^32^. It has been suggested that *Campylobacter* can survive under strong acid-shock conditions, and this is linked with the increased transcription of a subset of flagellar biosynthetic genes and stress responses, as well as the downregulation of genes involved in cell division and metabolism^33^. The flagellum not only has a distinct function in bacterial motility and cell binding, but also acts as a type III secretion system (T3SS)^9,34^. Flagellin is a potent activator of a broad range of host cell types involved in innate and adaptive immunity^35^. Rizal et al.^17^ examined the presence of the *fla*A factor in *C. jejuni* and *C. coli* derived from chicken and human isolates and obtained a 100% prevalence. Other authors have examined the presence of factors in *C. jejuni* and *C. coli* derived from humans, poultry meat, broiler, and bovine feces and obtained similar results^24^. Datta et al*.*^27^ also determined this factor in a group of 111 *C. jejuni* strains isolated from different sources and found that all of them were positive. Previous reports in Brazil have indicated that *fla*A is present in approximately 80% of *Campylobacter* spp. isolates from human and poultry samples^30,36^.

Another virulence gene examined in this study was *cad*F, one of the markers determining the adherence of *Campylobacter*. This gene was present in 77.9% of the isolates analyzed, similarly to previously reported results^37–39^. Previous reports in Japan, Australia, India, Poland, and Brazil have indicated that *cad*F is present in almost all *C. jejuni* isolates from poultry samples^17,24,27,30,35^. This gene encodes an adhesion factor used by *C. jejuni* to attach and, eventually, invade mammalian cells by binding to fibronectin, a component of the extracellular matrix^34,35^. Investigations reported that Δ*cad*F mutant strains were not able to colonize the gut of chickens^40^. Monteville et al*.*^23^ verified that the adhesion and transmigration of Δ*cad*F mutant strain to human INT-407 cells was reduced by 50%, when compared to a wild-type strain. Thus, the *cad*F gene, which appears to be essential for chicken gut colonization, may presumably have a similar role in the pathogenesis of human infection^41^. This gene is also an important mediator of material and information transfers between cells and their environment, and between compartments within cells. These surface-exposed proteins are conserved in *C. jejuni* strains and are also highly immunogenic in chicks^42^.

A bacterial toxin, CDT, may potentially play a role in disease development. This cytotoxin arrests eukaryotic cells to the G2 phase of the cell cycle, preventing them from entering mitosis, and leading to cell death. The toxin was named according to the morphological changes associated with its cytoplasmic distension action and its function is well documented^8,43^. In the present study, 74.3% of the *C. jejuni* strains carried the *cdt* complex. Datta et al.^27^ detected a 100% frequency of these genes in their studied samples, which included chicken feces, cattle feces, and human clinical samples. Rozynek et al.^41^ found a 100% prevalence in isolates from broiler carcasses. More than 76% of samples isolated from different sources were positive for *cdt*A, *cdt*B, and *cdt*C^24^. Previous studies have demonstrated extremely high rates of occurrence of this complex in *C. jejuni* isolated from several sources, including humans and poultry^35,44^. In Brazil, there are few studies on CDT in *C. jejuni*. Carvalho et al.^45^ stated that only 36.4% of *C. jejuni* samples from Brazilian broiler carcasses contained the CDT complex. In contrast, Melo et al*.*^30^ showed that almost all *Campylobacter* strains presented the *cdt* complex.

A significant percentage of *C. jejuni* carries the *vir*B11 gene localized on the pVir plasmid. The pVir plasmid encodes several genes that are homologous to a type IV secretion system and contributes to the ability of *Campylobacter jejuni* subsp. *jejuni* 81–176 to invade INT–407 cells in vitro, a marker that was correlated with virulence in a diarrheal disease ferret model^25,46^. Moreover, other studies showed both an association^47^ and a lack of association^48^ between the pVir plasmid and bloody diarrhea in *C. jejuni* enteritis. We found this gene in 22.1% of *C. jejuni* isolates. The occurrence of *vir*B11 varies among studies. In 2008, Wieczorek and Osek^24^ detected this gene in 18.5% of strains. Ten years later, the same authors found this gene in less than 5% of strains^35^. A small percentage of this marker in poultry samples has also been reported by other authors^27,41^. The reasons underlying this significant variation are still unknown.

The *iam* gene is a genetic marker that is preferentially associated with adherence and invasion of HEp–2 cells in vitro^49^. There is a good correlation between the clinical presentation of diarrhea and the isolation of *Campylobacter* strains that adhere to and invade HEp–2 cells. However, the involvement and function of the *iam* marker in the process of campylobacteriosis have not been precisely explained^17,50^. In this study, the *iam* sequence was not detected in any of the investigated isolates. Rozynek et al.^41^ detected this gene in 53.8% of *C. jejuni* samples isolated from chickens. The presence of this molecular marker is not restricted to *C. jejuni*, but is also present in *C. coli* and *C. lari*. Wieczorek and Osek^24^ showed the presence of this gene in 66% of the investigated strains. It was found that the marker was predominant in *C. coli* (82.9% of the isolates were positive), whereas only 46.7% of *C. jejuni* strains possessed this gene. The variable occurrence depends not only on the origin, but also on the isolated *Campylobacter* species. The fact that in some invasive strains the gene was not identified using PCR supports the existence of important polymorphisms and high heterogeneity in the *iam* locus or suggests that other genetic markers of invasion may occur in different loci^45^. The genetic characterization of *C. jejuni* through whole-genome sequencing (WGS) would probably result in the detection of this marker. Previous studies have detected *iam* in 99% of *Campylobacter* strains, showing that WGS has a high discriminatory power for this genus^51^.

The *wla*N gene is involved in variations of the lipooligosaccharide structure, facilitating the avoidance of *C. jejuni* in the host immune system. Our results showed that this marker was present in 10.7% of the tested *C. jejuni* strains. According to the investigation by Datta et al.^27^, this gene was present in 4.7% of isolates from broiler feces and in 23.8% of poultry meat isolates. The presence of the gene can vary depending on the geographic area and on the high rate of polymorphisms within *C. jejuni* strains. Koga et al.^50^ reported that the genetic polymorphism of *C. jejuni* determines the reactivity of autoantibodies and the clinical presentation of the Guillain–Barré syndrome, possibly through the modification of the host molecule mimic. Importantly, these positive isolates may have a higher pathogenic potential, and therefore, a higher capacity to induce autoimmune disease in their hosts. A previous study showed a correlation between an increased capacity of cell invasion (in vitro and in vivo), and the presence of the *wla*N gene, since 80% of the strains that lack this gene presented a lower or a total lack of invasiveness^52^.

Table 4 summarizes the occurrence of virulence markers among species (human, poultry, swine, and cattle). The relative frequencies of virulence genes observed in *C. jejuni* strains in the present study were similar or slightly lower than those observed in other species. These results indicate that strains isolated from poultry sources are potential carriers of virulence genes to humans.

**Table 4:** Relative Frequencies (%) of virulence markers in *Campylobacter jejuni* strains isolated from several sources found in other publications compared to the results of this current study.

Virulence markers	Source of isolation											
	Human				Cattle			Swine	Poultry			
	^31^	^57^	^46^	^58^	^46^	^58^	^59^	^59^	^31^^a^	^46^	^30^^b^	This study
*cdt*A	100	90	–	–	–	–	100	100	96.2	–	–	85
*cdt*B	100	90	100	96.1	100	96.7	100	100	98.1	100	–	80
*cdt*C	100	90	–	–	–	–	100	80	96.8	–	–	92.1
*cdt*ABC	–	88.7	–	–	–	–	86.7	80	–	–	97.7	74.3
*cad*F	100	97.9	–	98	–	81.4	100	100	98.7	–	97.7	77.9
*fla*A	100	–	–	–	–	–	100	100	98.7	–	84.1	78.6
*vir*B11	4.5	–	3.6	2	0	16.7	6.7	0	0	7.3	–	22.1
*wla*N	17.4	–	–	–	–	–	–		12.5	–	–	10.7
*iam*	15.5	4	–	98	–	81.4	–		8.9	–	–	0

^a^Results for strains isolated from poultry carcasses only.

^b^Results for strains isolated between 2015 and 2016 only.

It is important to note that the genetic characterization of *C. jejuni*, which is based on the molecular detection of genes, corresponds to a qualitative analysis. Thus, it is not possible to establish a linear relationship between the presence of the genes and in vivo virulence. However, molecular tools for virulence marker detection are essential to determine the genetic virulence profile of the strains circulating in poultry establishments and, thus, to determine specific control measures to mitigate the risks of dissemination. It is noteworthy that this study did not perform a temporal analysis of the data, and it is not known if the relative differences are stable over time or are restricted to the time of sample collection.

Brazil has an important position in the global trade market of chicken meat. However, unlike the European Union and United States, there is no specific legislation in the country for the analysis of *Campylobacter*. Moreover, campylobacteriosis is still underreported, and epidemiological and molecular studies on the diversity and virulence of *C. jejuni* are still few in the country^29,53^. Our results reinforce the urgent need for specific legislation adoption to prevent the spread of potentially pathogenic strains through the consumption of poultry products.

In conclusion, the present study revealed that eight potential pathogenicity genes of *C. jejuni* circulate in slaughter lines, among other poultry sources in Southern Brazil. The frequency of *C. jejuni* virulence markers, their relationship with clinical severity in humans, and the expression of virulence factors should be further investigated. The results provide more insight into factors related to the occurrence of this pathogen and in the understanding of their epidemiology.

## Methods

### Bacterial strains and growth conditions

This research was carried out in federally inspected slaughterhouses from five companies (A, B, C, D, and E) in Southern Brazil. A total of 20 visits were made to the processing plants. All selected establishments were representative of slaughterhouses located in the region; broilers were processed for production, with an average of 250,000 birds slaughtered per day, each having a standard live weight of 2.0 kg (4.4 lb). The processing line was operated under standard commercial conditions. In total, 980 samples were collected from swabs (cloacal and transportation boxes), water from the chiller tank, and carcasses throughout the slaughter line, as previously described by Borges et al*.*^53^. The samples were stored in isothermal ice boxes and taken to the laboratory for identification.

Buffered peptone water (BPW) (400 mL, 0.1%) was previously added to each carcass and manually shaken in a vigorous way for 1 min. An aliquot (1 mL) of each sample (swabs, water, and carcasses) was homogenized in Bolton broth (9 mL) supplemented with antimicrobials (cefoperazone 20 mg/L, vancomycin 20 mg/L, trimethoprim 20 mg/L, cycloheximide 50 mg/L; SR0183, Oxoid) and incubated under microaerophilic conditions using a gas tank with a mixture (10% CO_2_, 5% O_2_, and 85% N_2_) for 48 h at a temperature of 41.5 °C. After incubation, the suspension (100 µL) was spread onto an acetate mem deoxycholate agar (brane having 0.65-µm pores and placed on the surface of a modified charcoal cefoperazone mCCDA) plate with selective supplements (cefoperazone, 32 mg/L; amphotericin B, 10 mg/L; SR0155, Oxoid) for 30 min. The direct application of membrane filters to the surface of the plating media has been used to exclude unwanted microflora for the selective isolation of *Campylobacter*^54^. Due to its reduced pores, this membrane promotes the filtration of the suspension and provides a reduction of the bacterial contamination. The plate was incubated under microaerophilic conditions at 41.5 °C for 48 h. *Campylobacter* spp. suspected colonies (grayish, often with a metallic sheen, flat, and moist, with a tendency to spread)^31^ were examined for cell morphology by using phase-contrast microscopy. Subsequently, the isolates were purified using blood agar (BA) plates supplemented with 5% commercial sterile defibrinated sheep blood (Laborclin). Single colonies were picked and streaked onto BA plates, and preliminarily characterized based on their catalase reaction ability to hydrolyze hippurate and indoxyl acetate. The colonies were collected and suspended in ultrapure water (1 mL), transferred to a microtube, and frozen at − 20 °C. Isolates were stored in glycerol 15% at -80 °C. The species were determined using PCR^55^.

### DNA extraction

The template DNA for PCR was extracted using an adapted protocol described by Borsoi et al.^56^. Bacterial culture (1 mL) was boiled at 95 °C for 10 min. After centrifugation at 8000×*g* for 2 min, the supernatants were stored at − 20 °C and used as template DNA. The isolates were confirmed by using PCR based on the detection of the 16S rRNA and *map*A genes^55^.

### PCR primer design and amplification

The confirmed *Campylobacter jejuni* isolates were screened for the presence of eight pathogenic genes: *fla*A, *cad*F, *iam*, *vir*B, *wla*N, *cdt*A, *cdt*B, and *cdt*C. The primers, PCR conditions, and the length of the products generated in this study are listed in Table 5. The PCR conditions were adapted from previous studies. All PCR amplifications were performed in a mixture (25 µL) consisting of 10 × PCR Buffer [3 µL; 200 mM Tris–HCl (pH 8.4), 500 mM KCl], Taq thermostable DNA polymerase (0.3 µL, 5 U/µL), MgCl_2_ (1.2 µL, 25 mM), dNTPs (2.5 µL, 2.5 mM), extracted template DNA (2 µL) and each of the primers (0.5 µL each, 10 pmol/L). Sterile Milli-Q water was added in sufficient quantity to achieve a volume of 25 µL. All amplification reactions were performed in a thermal cycler. The cycles were performed as described in Table 2. For the visualization of the PCR products, 10 µL aliquots were subjected to electrophoresis in a 2% agarose gel in Tris–acetylated EDTA (TAE) buffer. DNA bands were stained with ethidium bromide for 2 h at 100 V, viewed under an ultraviolet transilluminator, and photographed. The size of the PCR amplicons was compared to that of the 100 bp DNA ladder.

**Table 5:** List of primers and PCR conditions used in this study.

Target gene	Primers	Sequence (5′ → 3′ʼ)	PCR conditions	Product (bp)	Reference
16S-rRNA	MD16S1 MD16S2	ATCTAATGGCTTAACCATTAAAC GGACGGTAACTAGTTTAGTATT	95 °C/10 min, 35 cycles: 95 °C/30 s, 59 °C/90 s, 72 °C/1 min, and 72 °C/10 min	857	*Campylobacter* genus identification^55,60^
*map*A	MDmapA1 MDmapA2	CTATTTTATTTTTGAGTGCTTGTG GCTTTATTTGCCATTTGTTTTATTA		589	*C. jejuni* species identification^55^
*fla*A	flaAF flaAR	GGATTTCGTATTAACACAAATGGTGC CTGTAGTAATCTTA AACATTTTG	94 °C/5 min, 30 cycles: 94 °C/1 min, 48 °C/1 min, 72 °C/1 min, and 72 °C/5 min	1700	http://campynet.vetinst.dk/Fla.htm
*cad*F	F2B R1B	TGGAGGGTAATTTAGATATG CTAATACCTAAAGTTGAAAC	94 °C/5 min, 30 cycles: 94 °C/1 min, 54 °C/1 min, 72 °C/1 min, and 72 °C/5 min	400	^33^
*iam*	IAMF IAMR	GCGCAAAATATTATCACCC TTCACGACTACTATGCGG	94 °C/5 min, 30 cycles: 94 °C/1 min, 55 °C/1 min, 72 °C/1 min, and 72 °C/5 min	518	^49^
*vir*B11	virBF virBR	GAACAGGAAGTGGAAAAACTAGC TTCCGCATTGGGCTATATG	95 °C/5 min, 35 cycles: 95 °C/30 s, 53.5 °C/30 s, 72 °C/30 s, and 72 °C/5 min	708	^25^
*wla*N	wlaN-DL39 Cj1139cF	TTAAGAGCAAGATATGAAGGTG TGCTGGGTATACAAAGGTTGTG	95 °C/10 min, 25 cycles: 95 °C/30 s, 60 °C/30 s, 72 °C/1 min, and 72 °C/5 min	434	^54^
*cdt*A	cdtAF cdtAR	CCTTGTGATGCAAGCAATC ACACTCCATTTGCTTTCTG	94 °C/5 min, 30 cycles: 94 °C/1 min, 49 °C/30 s, 72 °C/1 min, and 72 °C/5 min	370	^60^
*cdt*B	cdtBF cdtBR	CAGAAAGCAAATGGAGTGTT AGCTAAAAGCGGTGGAGTAT	94 °C/5 min, 30 cycles: 94 °C/1 min, 51 °C/30 s, 72 °C/1 min, and 72 °C/5 min	620	^27^
*cdt*C	cdtCF cdtCR	CGATGAGTTAAAACAAAAAGATA TTGGCATTATAGAAAATACAGTT	94 °C/5 min, 30 cycles: 94 °C/1 min, 47 °C/30 s, 72 °C/1 min, and 72 °C/5 min	182	^27^

### Statistical analysis

The obtained data were subjected to statistical analysis using the PASW Statistics software (IBM, Hong Kong). Descriptive statistics (frequency distribution) was used to determine the presence of the virulence genes according to the source and the poultry company from which the sample was isolated. Chi-square (χ^2^) and Fisher’s exact tests were used to compare the frequencies of virulence-associated genes among each other and among sources and companies from which samples were isolated. Statistical significance was defined at a *p* < 0.05, and Bonferroni correction was applied to adjust confidence intervals for multiple hypothesis testing. Adjusted *p*-values are indicated at each Table.

## Data Availability

The datasets generated during and/or analysed during the current study are available from the corresponding author on reasonable request.
